# Efficacy of albumin-bound paclitaxel versus paclitaxel in esophageal cancer: a systematic review and meta-analysis

**DOI:** 10.3389/fonc.2025.1612678

**Published:** 2025-08-18

**Authors:** Jiayang Song, Yu Xiong, Hao Liu, Wei Zhang, Zhongfu Tan, Nanjiang Liu

**Affiliations:** ^1^ Department of Pharmacy, Cangxi People’s Hospital, Guangyuan, Sichuan, China; ^2^ Department of Pharmacy, Affiliated Hospital of University of Electronic Science and Technology of China Medical School·Mianyang Central Hospital, Mianyang, Sichuan, China; ^3^ National Health Commission Key Laboratory of Nuclear Technology Medical Transformation, Mianyang, Sichuan, China; ^4^ Science and Technology Department of North Sichuan Medical College, Nanchong, Sichuan, China; ^5^ Department of Pharmacy, Kaizhou District People’s Hospital, Kaizhou, Chongqing, China

**Keywords:** albumin-bound paclitaxel, paclitaxel, esophageal cancer, efficacy, adverse reactions, systematic review, meta-analysis

## Abstract

**Objective:**

To systematically evaluate the efficacy and safety of albumin-bound paclitaxel and paclitaxel in the treatment of esophageal cancer.

**Methods:**

Seven databases (PubMed, Cochrane Library, Embase, China National Knowledge Infrastructure, Wanfang Data, China Science and Technology Journal Database, and China Biology Medicine disc) were searched for randomized controlled trials (RCTs) of albumin-bound paclitaxel and paclitaxel in the treatment of esophageal cancer. The search was conducted from database inception to February 2025. Literature quality was assessed using the Cochrane Risk of Bias Tool version 1 (RoB 1), and the systematic review and meta-analysis was performed using RevMan 5.4.1 and STATA18.

**Results:**

Eleven RCTs were included. Meta-analysis demonstrated that albumin-bound paclitaxel significantly improved objective response rate [ORR; relative risk (RR) = 1.67, 95% confidence interval (CI) [1.45, 1.92], p < 0.001, I^2^ = 0%] and disease control rate (DCR; RR = 1.69, 95% CI [1.43, 1.98], p < 0.001, I^2^ = 0%) compared to paclitaxel. It also showed superior improvements in serum tumor markers: cancer antigen 125 (CA125) [mean difference (MD) = −1.69, 95% CI [−2.73, −0.65], p < 0.001, I^2^ = 83%], Carbohydrate antigen 19-9 (CA199) (MD = −2.12, 95% CI [−3.39, −0.84], p = 0.001, I^2^ = 85%), and carcinoembryonic antigen (CEA) (MD = −2.01, 95% CI [−2.53, −1.50], p < 0.001, I^2^ = 99%), although Squamous Cell Carcinoma Antigen (SCC) improvement was non-significant (MD = −1.19, 95% CI [−2.61, 0.24], p > 0.001, I^2^ = 100%). Regarding safety, albumin-bound paclitaxel had markedly lower incidences of diarrhea (RR = 0.49, 95% CI [0.33, 0.72], p = 0.003, I^2^ = 0%), nausea/vomiting (RR = 0.61, 95% CI [0.46, 0.80], p < 0.001, I^2^ = 0%), thrombocytopenia (RR = 0.61, 95% CI [0.44, 0.85], p = 0.004, I^2^ = 22%), and myalgia/arthralgia (RR = 0.45, 95% CI [0.22, 0.94], p = 0.03, I^2^ = 0%), while neutropenia showed no significant difference (RR = 0.58, 95% CI [0.32, 1.03], p = 0.006, I^2^ = 0%).

**Conclusion:**

Compared to paclitaxel, albumin-bound paclitaxel (nab-paclitaxel) demonstrates superior efficacy in the treatment of esophageal cancer, with fewer adverse reactions such as diarrhea, thrombocytopenia, and musculoskeletal pain.

## 1 Introduction

Esophageal cancer ranks seventh in incidence and sixth in mortality among all cancers (1). In China, esophageal cancer ranks eighth in terms of incidence rate and fifth in terms of mortality rate. The incidence of esophageal cancer is relatively insidious, and when it is detected, it is often in the middle or late stage of cancer, with a poor prognosis (2). Neoadjuvant chemotherapy or chemoradiotherapy remains the standard treatment for resectable esophageal cancer. Chemotherapy or chemotherapy combined with targeted therapy and immunotherapy has become a prevalent approach for patients with advanced esophageal cancer. Chemotherapy is very important in the treatment of esophageal cancer, and common chemotherapy regimens include fluorouracil combined with platinum, and paclitaxel combined with platinum (3, 4). Taxanes, including paclitaxel and docetaxel, have emerged as common chemotherapeutic agents in the management of esophageal cancer and have been developed in various formulations, such as liposomes, nanoparticles, and polymeric micelles (5–7). Albumin-bound paclitaxel employs nanoparticle albumin-bound technology to bind paclitaxel to human serum albumin (HSA), thereby enhancing the bioavailability and addressing the poor aqueous solubility of paclitaxel. Additionally, it avoids the allergic reactions caused by the use of polyoxyethylene castor oil (Cremophor EL). Thus, albumin-bound paclitaxel has the advantages of better tumor delivery rates and reduced need for pretreatment (8).

There is a clear distinction between the use of albumin-bound paclitaxel and paclitaxel in the chemotherapy regimen of “Esophageal Cancer Diagnosis and Treatment Guidelines 2024” by the Chinese Society of Clinical Oncology (CSCO). There is a lack of large-sample studies on efficacy and toxicity profiles of the two drugs, yet albumin-bound paclitaxel and paclitaxel are used interchangeably in clinical practice. This study systematically evaluates the efficacy and safety of albumin-bound paclitaxel and paclitaxel in esophageal cancer treatment using evidence-based medicine, aiming to provide a basis for clinical decision-making.

## 2 Materials and methods

### 2.1 Inclusion criteria and exclusion criteria

#### 2.1.1 Study design

The types of studies included were all publicly published clinical randomized controlled trials (RCTs), which were original peer-reviewed Chinese or English papers.

#### 2.1.2 Research subjects

All the included patients were diagnosed with esophageal cancer (determined by cytology, pathology, and imaging), and there was no restriction on gender, race, region, or the course of the disease.

#### 2.1.3 Interventions

Clinical studies were divided into the experimental group and the control group. The experimental group received albumin-bound paclitaxel with or without platinum-based drugs. The dosage of albumin-bound paclitaxel was 150–270 mg/m^2^ every 3 weeks or split into weekly regimens. The control group received paclitaxel combined with or without platinum-based drugs. The dosage of paclitaxel was 125–200 mg/m^2^ every 3 weeks.

#### 2.1.4 Outcome measures

The main outcome measures of this study included the following: 1) response rate, including objective response rate (ORR) and disease control rate (DCR), with ORR = CR [complete response (CR)] + PR [partial response (PR)], and DCR = CR + PR + SD [stable disease (SD)] (9); 2) CA125; 3) CA199; 4) CEA; 5) SCC; 6) nausea and vomiting; 7) thrombocytopenia; 8) neutropenia; 9) diarrhea; and 10) musculoskeletal pain.

#### 2.1.5 Exclusion criteria

The exclusion criteria were as follows: 1) non-randomized controlled trials, 2) repeated published literature, and 3) literature for which outcome indicators are not directly or computationally available.

### 2.2 Literature retrieval strategy

Seven databases were searched, comprising PubMed, Cochrane Library, Embase, China National Knowledge Infrastructure (CNKI), Wanfang Data, China Science and Technology Journal Database (VIP), and China Biology Medicine disc (CBMdisc). The search was conducted from database inception to February 2025. The search strategy involved a combination of title and abstract. Search terms included “albumin-bound paclitaxel”, “paclitaxel”, and “esophageal neoplasms” (the retrieval strategy is detailed in the **Supplementary Material**).

### 2.3 Literature screening and data extraction

Two researchers independently screened the literature according to the inclusion and exclusion criteria. First, the titles and abstracts were read to preliminarily exclude the articles that did not meet the criteria. Then, the full texts were further read through to finalize the literature to be included. Two authors extracted the following data into a pre-designed Excel file and recorded it: the first author’s name, publication year, whether allocation concealment was used, whether blinded, gender, age, sample size, intervention time, and outcome measures. If there was a disagreement between the two authors, a third author would review and decide whether to include the study.

### 2.4 Quality evaluation

Literature quality was assessed using the Cochrane Risk of Bias Tool version 1 (RoB 1), which included the following: random sequence generation, allocation concealment, blinding of participants and personnel, blinding of outcome assessment, incomplete outcome data, selective reporting, and other bias. The above seven items were categorized into “low risk of bias”, “high risk of bias”, and “unclear risk of bias” with an assessment (10).

### 2.5 Statistical analysis

The systematic review and meta-analysis was performed using RevMan 5.4.1 software. For dichotomous variables, the results were expressed using relative risk (RR) with its corresponding 95% confidence interval (CI). For continuous variables, results should be presented as mean difference (MD) along with its 95% CI. For continuous variables, if the same outcome measures, measurement methods, and units of measurement were used, MD did not need to be standardized; otherwise, it should be standardized. Additionally, heterogeneity testing was performed on the included articles. Heterogeneity among the included studies was assessed. If the results showed no significant heterogeneity (p > 0.1 for the Q-test and I^2^ < 50%), a fixed-effects model was applied for the primary analysis, with results from a random-effects model also provided. Otherwise, a random-effects model was used. If heterogeneity was high, subgroup analyses were conducted to explore the causes. Subgroup analysis involved dividing all included studies into several subgroups based on one or more categorical covariate, conducting meta-analyses independently within each subgroup, calculating the pooled effect size and its confidence interval for each subgroup, comparing the pooled effect sizes between different subgroups, and using statistical tests to determine whether the differences between groups are statistically significant (11). Due to the differential chemosensitivity of tumors to chemotherapeutic agents across distinct tumor stages or treatment phases—which may contribute to variations in therapeutic efficacy—this study incorporated different treatment phases as a subgroup factor for subgroup analysis (12). When >10 articles were included, sensitivity analysis was performed using STATA18 to assess the robustness of the results, and contour-enhanced meta-analysis funnel plots and Egger’s regression test were used to analyze publication bias. This study used leave-one-out analysis for sensitivity analysis, sequentially excluding each study included in the meta-analysis and then re-conducting the combined analysis using the remaining studies.

## 3 Results

### 3.1 Literature retrieval results and characteristics of included literature

**Figure 1:**
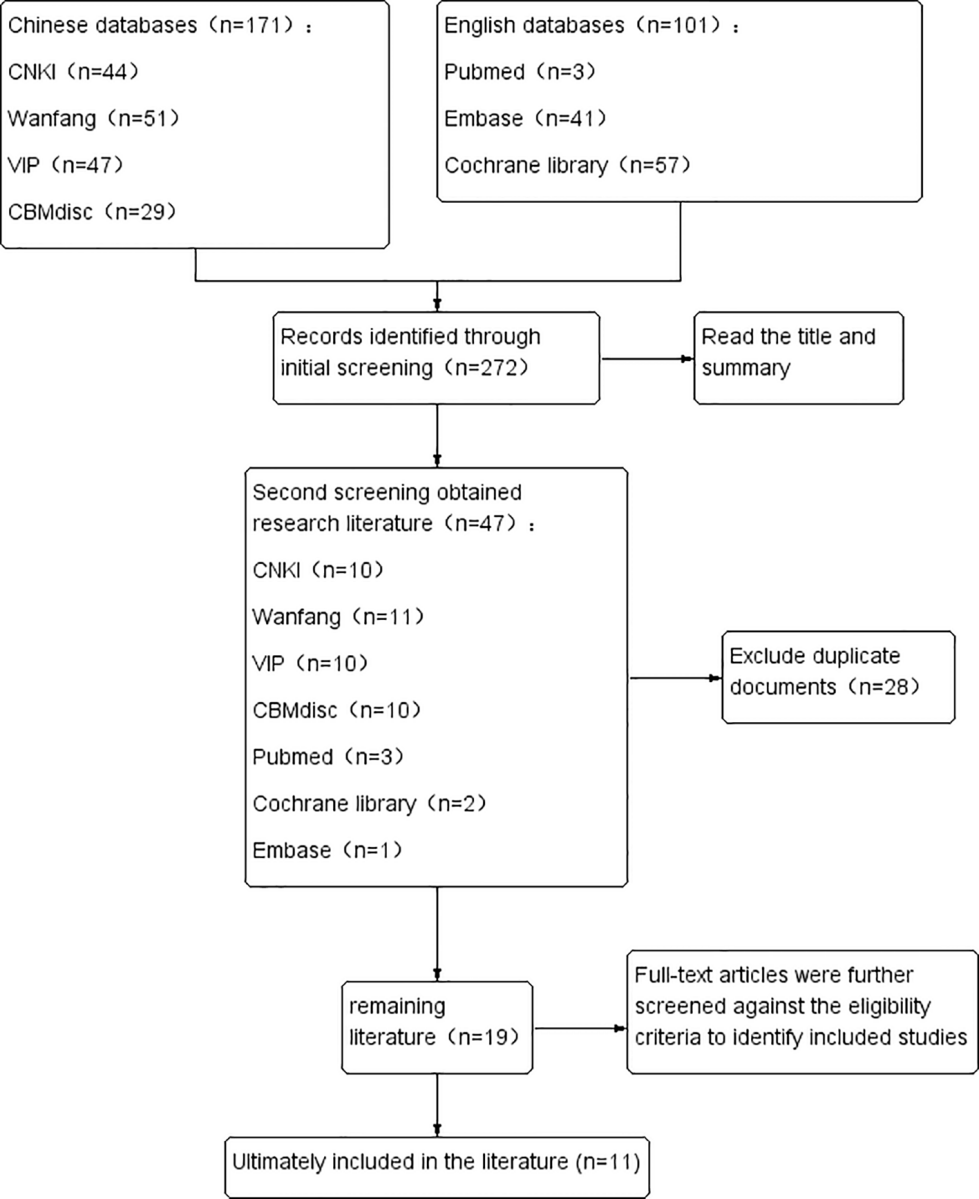
Literature screening flowchart.

A total of 272 articles were initially obtained. By screening the titles and abstracts, 47 studies comparing albumin-bound paclitaxel with paclitaxel for esophageal cancer were included. After excluding 28 articles due to not meeting the inclusion criteria, 19 articles were rescreened by reading the full texts, and six articles were further excluded due to not meeting the inclusion criteria. Finally, 11 articles were included in the analysis. The efficacy indicators of 11 literature sources were analyzed, and the efficacy and safety outcome indicators of literature sources with more than two articles were ultimately included. The literature screening process is shown in **Figure 1**, and the basic characteristics of the included articles are shown in **Table 1**.

**Table 1:** Basic characteristics of the included literature.

Included articles	Sample size		Gender (male/female)		Age (x ± s)/years		Interventions		Course	Treatment phase	Outcome measures
	Albumin-bound paclitaxel	Paclitaxel	Albumin-bound paclitaxel	Paclitaxel	Albumin-bound paclitaxel	Paclitaxel	Albumin-bound paclitaxel	Paclitaxel			
Dang, Shengqiang, 2021 (13)	31	31	16/15	17/14	58.4 ± 13.12	59.82 ± 8.77	150 mg/m^2^ + radiotherapy	150 mg/m^2^ + cisplatin 70 mg/m^2^ d1	Every 3 weeks, 2 cycles	Neoadjuvant therapy	①④⑤
Zhao, Hongdan, 2022 (14)	21	21	15/6	14/7	51.63 ± 6.67	51.35 ± 6.83	130 mg/m^2^ d1, 8+ nedaplatin 75 mg/m^2^ d1	175 mg/m^2^ + nedaplatin 75 mg/m^2^ d1	Every 3 weeks, 2 cycles	Advanced chemotherapy	①④⑥⑦⑨
Xing, Lei, 2024 (15)	33	33	22/11	20/13	54.70 ± 7.60	55.05 ± 7.30	125 mg/m^2^ d1, 8++ nedaplatin 40 mg/m^2^ d1~2	125~175 mg/m^2^ d1 + cisplatin 25 mg/m^2^ d1~3	Every 3 weeks, up to 6–8 cycles	Advanced chemotherapy	①②③④⑤⑥⑦⑧⑩
Jiang, Yong, 2019 (16)	30	30	15/15	18/12	64.75 ± 0.73	64.80 ± 0.69	260 mg/m^2^ d1 + cisplatin 25 mg/m^2^ d1~3	140 mg/m^2^ + cisplatin 25 mg/m^2^ d1~3	Every 3 weeks	Mid- to late-stage chemotherapy	①
Tu, Zhengjun, 2020 (17)	28	28	14/14	16/12	59.48 ± 1.92	58.03 ± 2.14	260 mg/m^2^ d1 + cisplatin 25 mg/m^2^ d1~3	140 mg/m^2^ + cisplatin 25 mg/m^2^ d1~3	Every 3 weeks	Advanced chemotherapy	①
Ren, Chao, 2020 (18)	30	30	18/12	20/10	54.41 ± 4.88	54.32 ± 4.96	260 mg/m^2^ d1 + cisplatin 75 mg/m^2^ d1~3	135 mg/m^2^ d1 + cisplatin 75 mg/m^2^ d1	Every 3 weeks	Advanced chemotherapy	①⑥⑦⑨
Zhong, Haijun, 2022 (19)	50	50	24/26	28/22	51.22 ± 6.30	51.36 ± 6.05	260 mg/m^2^ d1 + cisplatin 75 mg/m^2^ d1	140 mg/m^2^ d1 + cisplatin 75 mg/m^2^ d1	Every 3 weeks, 3–4 cycles	Neoadjuvant therapy	①②③④⑤⑥⑦⑧⑩
Sha, Ou, 2024 (20)	32	32	19/13	18/14	49.78 ± 9.87	48.98 ± 9.65	250 mg/m^2^ + radiotherapy	150 mg/m^2^ + cisplatin 70 mg/m^2^ d1	Every 3 weeks, 2 cycles	Neoadjuvant therapy	①②③④⑥⑦⑧
Yan, Jing, 2019 (21)	38	80	21/17	39/41	45.3 ± 4.9	47.3 ± 5.2	135 mg/m^2^ d1, 8+ cisplatin 20 mg/m^2^ d1~3	135~200 mg/m^2^ d1 + cisplatin 20 mg/m^2^ d1~3	Every 3 weeks, 2 cycles	Advanced chemotherapy	①⑥⑦⑧⑩
Wu, Yigen, 2024 (22)	35	35	21/14	20/15	58.15 ± 2.58	58.36 ± 2.71	130 mg/m^2^ d1, 8+ nedaplatin 75 mg/m^2^ d1	150 mg/m^2^ + nedaplatin 75 mg/m^2^ d1	Every 3 weeks, 3 cycles	Advanced chemotherapy	①②③④⑥⑨
Chen, Yuandi, 2023 (23)	24	24	16/8	15/9	52.77 ± 3.65	54.33 ± 3.57	130 mg/m^2^ d1, 8+ nedaplatin 80 mg/m^2^ d1	150 mg/m^2^ + nedaplatin 80 mg/m^2^ d1	Every 3 weeks, 1 cycle	Advanced chemotherapy	①③④⑤

Outcome measures: ①, efficacy; ②, CA125; ③, CA199; ④, CEA; ⑤, SCC; ⑥, nausea and vomiting; ⑦, thrombocytopenia; ⑧, granulocytopenia; ⑨, diarrhea; ⑩, musculoskeletal pain.

### 3.2 Methodological quality evaluation

**Figure 2:**
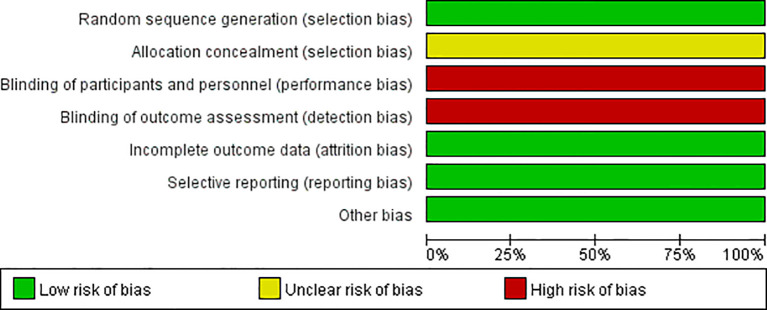
Risk of bias graph.

**Figure 3:**
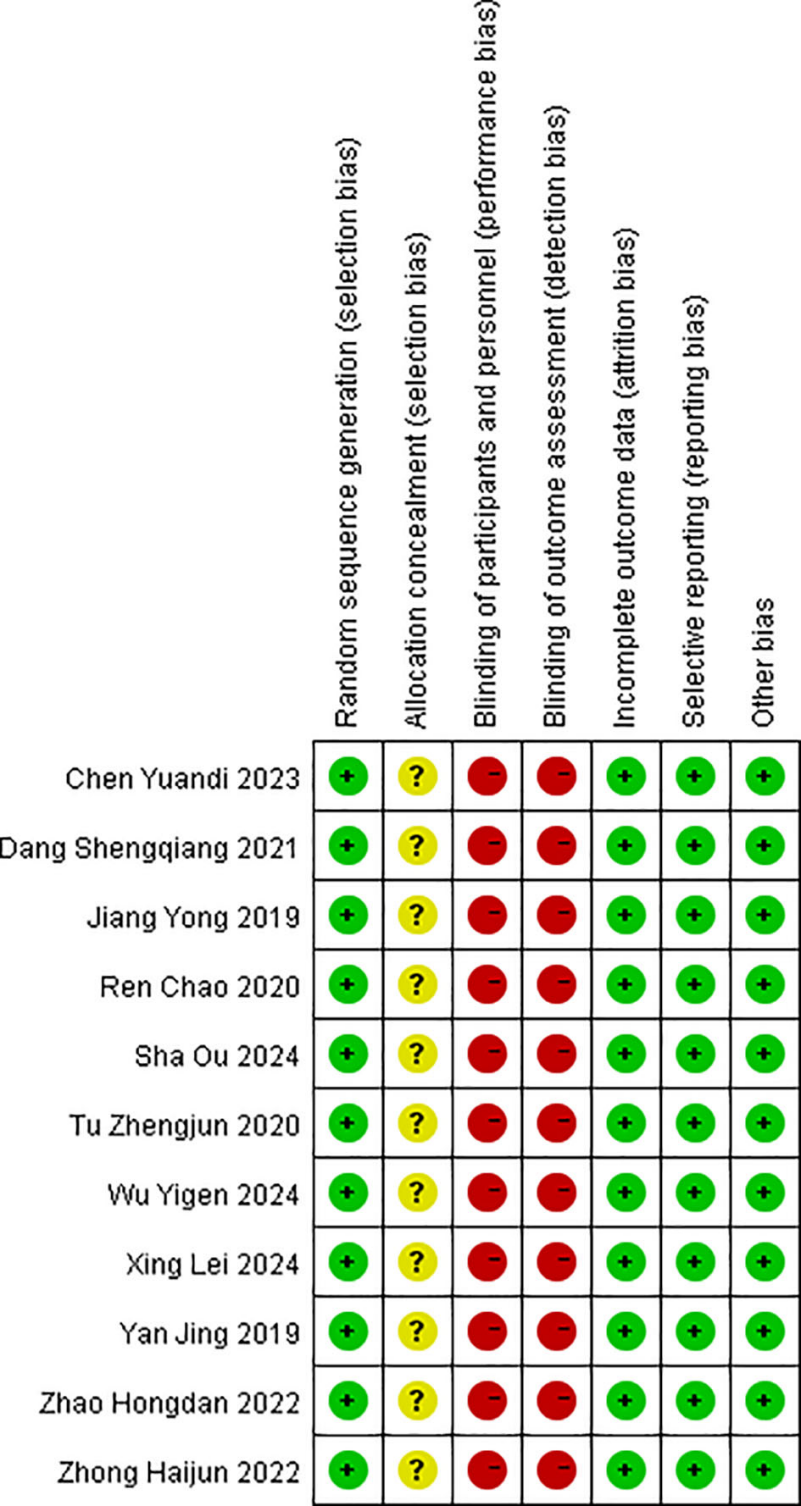
Risk of bias summary.

All 11 studies described the generation of random sequences; the allocation concealment was unclear in all studies; 11 studies explicitly obtained informed consent from patients, and the assessment of outcome measures was non-blinded by default. All 11 studies comprehensively reported outcome measures; none of the 11 studies selectively reported outcomes. Baseline characteristics were balanced across studies. The results of the risk of bias are shown in **Figures 2** and **3**.

### 3.3 Quality assessment of evidence

This study used the Grading of Recommendations, Assessment, Development and Evaluations (GRADE) approach to assess the quality of evidence. The grading results showed that the evidence quality ratings for ORR, DCR, diarrhea, nausea and vomiting, thrombocytopenia, and musculoskeletal pain were moderate. However, the evidence quality ratings for tumor markers CA125, CA199, CEA, and granulocytopenia were low, while the evidence quality rating for tumor marker SCC was very low. The results are shown in **Table 2**.

**Table 2:** GRADE evidence level assessment results.

Outcomes	Number of studies	RR or MD (95% CI)	Sample size	Certainty (quality)	Downgrade reasons
ORR	11	RR = 1.67 (1.45, 1.92)	352	Moderate	Risk of bias (−1)
DCR	8	RR = 1.69 (1.43, 1.98)	262	Moderate	Risk of bias (−1)
CA125	4	MD = −1.69 (−2.73, −0.65)	150	Low	Risk of bias (−1) Inconsistency (−1)
CA199	5	MD = −2.12 (−3.39, −0.84)	174	Low	Risk of bias (−1) Inconsistency (−1)
CEA	7	MD = −2.01 (−2.53, −1.50)	226	Low	Risk of bias (−1) Inconsistency (−1)
SCC	4	MD = −1.19 (−2.61, 0.24)	129	Very low	Risk of bias (−1) Inconsistency (−1) Imprecision (−1)
Diarrhea	3	RR = 0.49 (0.33, 0.72)	86	Moderate	Risk of bias (−1)
Nausea and vomiting	7	RR = 0.61 (0.46, 0.80)	239	Moderate	Risk of bias (−1)
Thrombocytopenia	6	RR = 0.61 (0.44, 0.85)	204	Moderate	Risk of bias (−1)
Granulocytopenia	4	RR = 0.58 (0.32, 1.03)	153	Low	Risk of bias (−1) Imprecision (−1)
Musculoskeletal pain	3	RR = 0.45 (0.22, 0.94)	121	Moderate	Risk of bias (−1)

GRADE, Grading of Recommendations, Assessment, Development and Evaluations; RR, relative risk; MD, mean difference; ORR, objective response rate; DCR, disease control rate.

### 3.4 Meta-analysis results

#### 3.4.1 Comparison of efficiency

**Figure 4:**
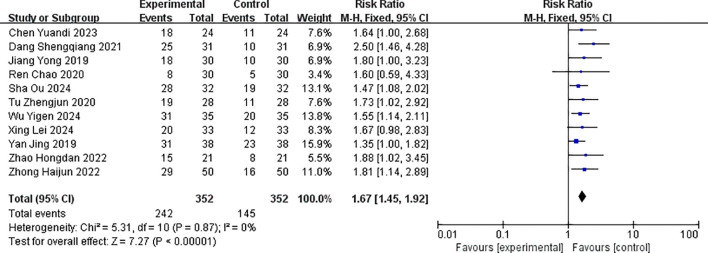
Comparison of objective response rates between albumin-bound paclitaxel group and paclitaxel group. The test group was albumin-bound paclitaxel group, and the control group was paclitaxel group, the same as below.

The efficiency of this study includes ORR and DCR. Some studies reported CR and PR, while others also reported SD. ORR and DCR can be obtained by adding them together. All 11 studies reported the objective response rate after treatment (14–23). There was no statistical heterogeneity among the outcomes (p = 0.87, I^2^ = 0%), and a fixed-effects model was used for pooling effect sizes. The meta-analysis results showed that the combined effect size RR = 1.67 (1.45, 1.92), which was statistically significant (Z = 7.27, p < 0.001). The result of the random-effects model was RR = 0.27 (0.21, 0.43), which was statistically significant (Z = 8.10, p < 0.001), and there was no statistical heterogeneity between studies (p = 0.71, I^2^ = 0%). This suggested that the objective response rate in the albumin-bound paclitaxel group was significantly higher than that in the paclitaxel group, with the objective response rate in the albumin-bound paclitaxel group being 1.67 times that of the paclitaxel group (p < 0.05). The forest plot of the objective response rate is shown in **Figure 4**.

**Figure 5:**
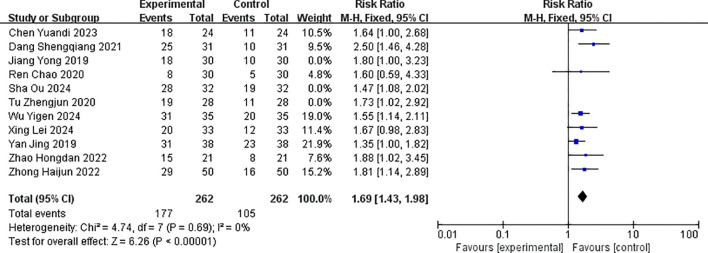
Comparison of disease control rates between albumin-bound paclitaxel group and paclitaxel group.

Eight studies reported disease control rates after treatment (14–19, 21–23). There was no statistical heterogeneity among the outcomes (p = 0.69, I^2^ = 0%), and fixed effects were used for pooling effect sizes. The meta-analysis results showed that the combined effect size RR = 1.69 (1.43, 1.98), which was statistically significant (Z = 6.26, p < 0.001). The result of the random-effects model was RR = 0.27 (0.20, 0.35), which was statistically significant (Z = 6.89, p < 0.001), and there was no statistical heterogeneity between studies (p = 0.41, I^2^ = 3%). This suggested that the disease control rate in the albumin-bound paclitaxel group was significantly higher than that in the paclitaxel group, with the objective response rate in the albumin-bound paclitaxel group being 1.67 times that of the paclitaxel group (p < 0.05). The forest plot of the disease control rate is shown in **Figure 5**.

#### 3.4.2 Comparison of serum tumor markers

In this meta-analysis, there were four, five, seven, and four papers that reported the four serum markers CA125, CA199, CEA, and SCC, respectively, and all of them did not have statistical significance of the baseline difference of serum markers between the two groups of patients prior to the treatment, so this meta-analysis directly counts and compares the results of the post-treatment period.

Four studies reported the post-treatment values of serum tumor marker CA125 (15, 19, 20, 22). There was statistical heterogeneity among the studies (p < 0.001, I^2^ = 83%), and a random-effects model was used for pooling effect sizes. The meta-analysis results showed that the combined effect size MD = −1.69 (−2.73, −0.65), which was statistically significant (Z = 3.19, p < 0.001). This indicated that the reduction of CA125 in the albumin-bound paclitaxel group was significantly higher than that in the paclitaxel group, with an increase of 1.65 in the albumin-bound paclitaxel group compared to the paclitaxel group (p < 0.05).

**Figure 6:**
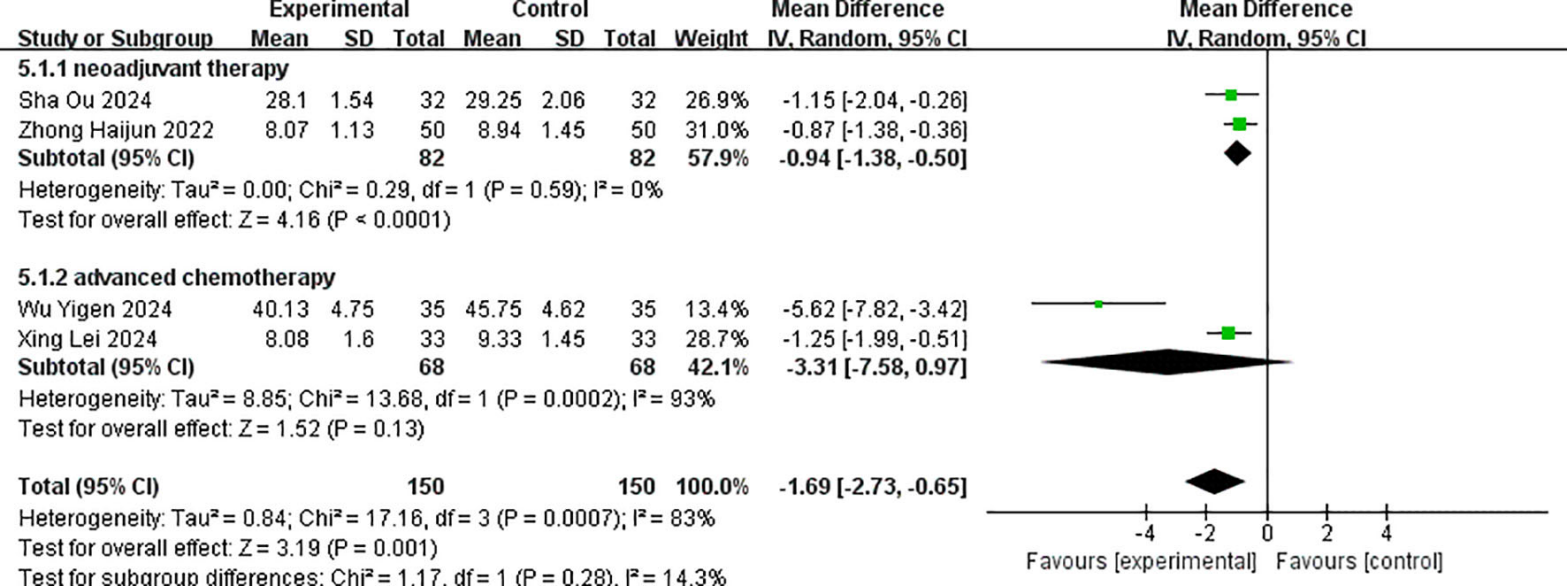
Comparison of the decline of CA125 between albumin-bound paclitaxel group and paclitaxel group.

Subgroup analysis was performed according to different treatment stages. The results showed that the combined effect size MD = −0.94 (−1.38, −0.50), which was statistically significant (Z = 4.16, p < 0.001) for the neoadjuvant therapy group. This indicated that the decrease of CA125 in the albumin-bound paclitaxel group was significantly higher than that in the paclitaxel group. For the advanced treatment group, the combined effect size MD = −3.31 (−7.58, 0.97), which was not statistically significant (Z = 1.52, p = 0.13), suggesting no difference in the improvement of CA125 between the albumin-bound paclitaxel group and the paclitaxel group. The forest plot for the improvement in CA125 after treatment is shown in **Figure 6**.

Five studies reported the post-treatment values of serum tumor marker CA199 (15, 19, 20, 22, 23). There was statistical heterogeneity among the studies (p < 0.001, I^2^ = 85%), and a random-effects model was used to pool effect sizes. The meta-analysis results showed that the combined effect size MD = −2.12 (−3.39, −0.84), which was statistically significant (Z = 3.26, p = 0.001). This indicated that the decrease of CA199 levels in the albumin-bound paclitaxel group was significantly higher than that in the paclitaxel group, with an increase of 2.12 in the albumin-bound paclitaxel group compared to the paclitaxel group (p < 0.05).

**Figure 7:**
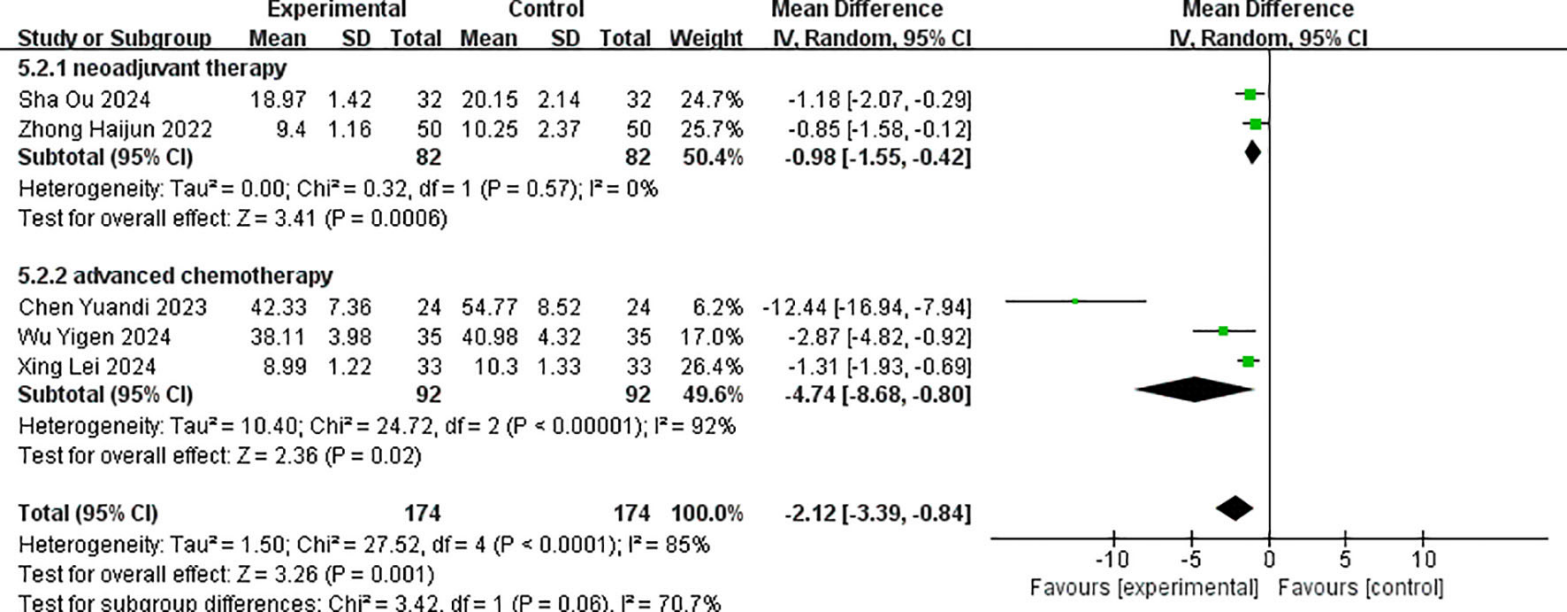
Comparison of the decline of CA199 between albumin-bound paclitaxel group and paclitaxel group.

Subgroup analysis was conducted according to different treatment stages, and the results showed that the combined effect size MD = −0.98 (−1.55, −0.42), which was statistically significant (Z = 3.41, p < 0.001) for the neoadjuvant therapy group. This indicated that the decrease of CA199 in the albumin-bound paclitaxel group was significantly higher than that in the paclitaxel group. For the advanced treatment group, the value of the combined effect MD was −4.74 (−8.68, −0.80), which was also statistically significant (Z = 2.36, p = 0.02), suggesting that the improvement in CA199 in the albumin-bound paclitaxel group was better than that in the paclitaxel group. The forest plot for the improvement in CA199 after treatment is shown in **Figure 7**.

Seven studies reported the post-treatment values of serum tumor markers CEA (13–15, 19, 20, 22, 23). There was statistical heterogeneity among the studies (p < 0.001, I^2^ = 99%), and a random-effects model was used for pooling effect sizes. The meta-analysis results showed that the combined effect size MD = −2.01 (−2.53, −1.50), which was statistically significant (Z = 7.62, p < 0.001). This indicated that the decrease of CEA in the albumin-bound paclitaxel group was significantly higher than that in the paclitaxel group, with an increase of 2.01 in the albumin-bound paclitaxel group compared to the paclitaxel group (p < 0.05).

**Figure 8:**
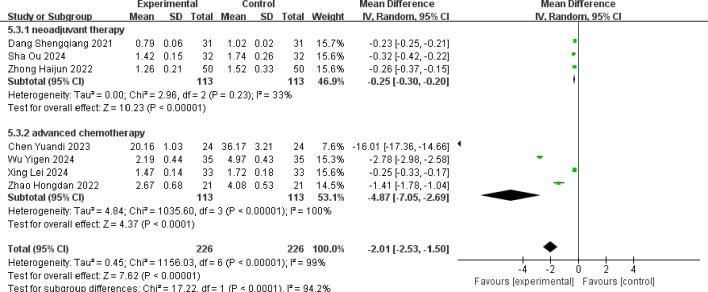
Comparison of the decline of CEA between albumin-bound paclitaxel group and paclitaxel group.

Subgroup analysis was performed according to different treatment stages, and the results showed that the combined effect size MD = −0.25 (−0.30, −0.20), which was statistically significant (Z = 10.23, p < 0.001) for the neoadjuvant therapy group, indicating that the reduction of CEA in the albumin-bound paclitaxel group was significantly higher than that in the paclitaxel group. For the advanced treatment group, the combined effect size MD = −4.87 (−7.05, −2.69), with statistical significance (Z = 4.37, p < 0.001), suggesting that the improvement in the CEA reduction of the albumin-bound paclitaxel group was better than that in the paclitaxel group. The forest plot showing the improvement in CEA after treatment is shown in **Figure 8**.

Four studies reported the post-treatment values of serum tumor marker SCC (13, 15, 19, 23). There was statistical heterogeneity among the studies (p < 0.001, I^2^ = 100%), and a random-effects model was used for pooling effect sizes. The meta-analysis showed that the combined effect size MD = −1.19 (−2.61, 0.24), which was not statistically significant (Z = 1.63, p = 0.1). This revealed that the reduction of SCC in the albumin-bound paclitaxel group was not significantly different from that in the paclitaxel group.

**Figure 9:**
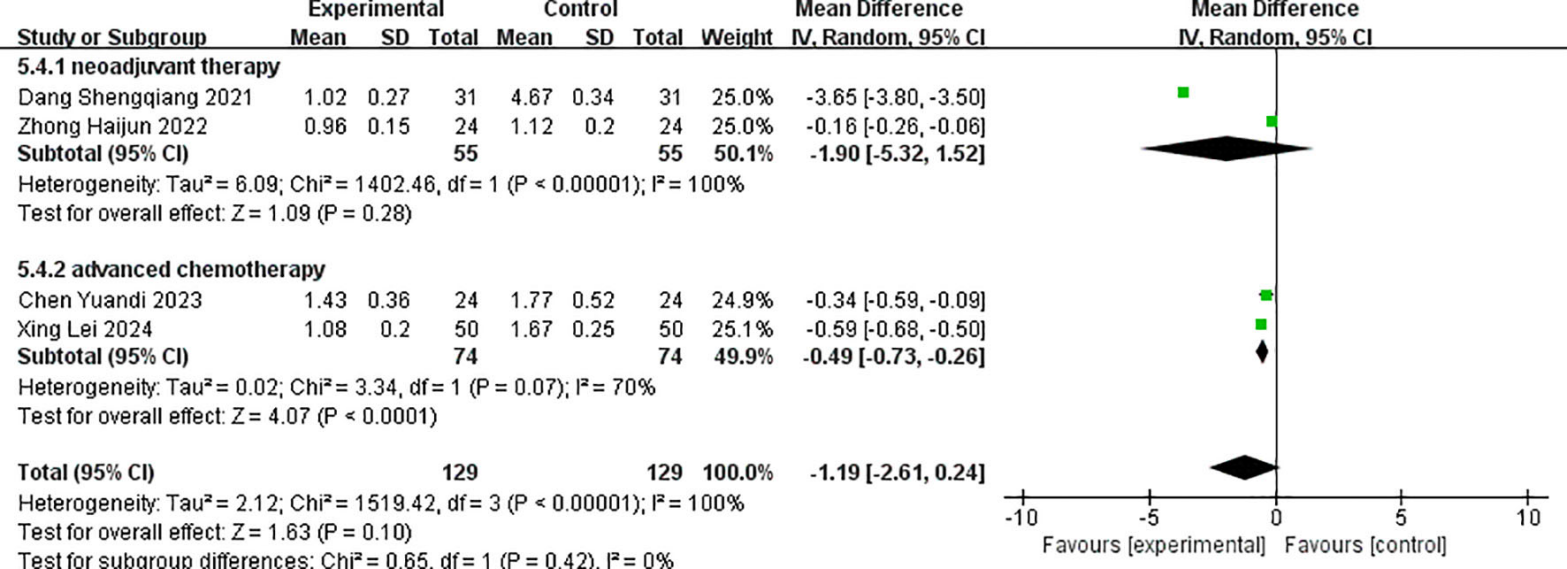
Comparison of the decline of SCC between albumin-bound paclitaxel group and paclitaxel group.

According to different treatment stages, subgroup analysis showed that for the neoadjuvant and advanced treatment groups, the reduction of SCC in the albumin-bound paclitaxel group was not significant compared with that in the paclitaxel group. The forest plot for SCC improvement after treatment is shown in **Figure 9**.

#### 3.4.3 Safety analysis

The literature included in this study analyzed diarrhea, nausea and vomiting, thrombocytopenia, granulocytopenia, and musculoskeletal pain (14, 15, 18–22). Meta-analysis showed that the heterogeneity of these adverse reactions was low (p = 0.46, I^2^ = 0%; p = 0.53, I^2^ = 0%; p = 0.27, I^2^ = 22%; p = 0.4, I^2^ = 0%; p = 0.54, I^2^ = 0%), and all were combined using a fixed-effects model.

**Figure 10:**
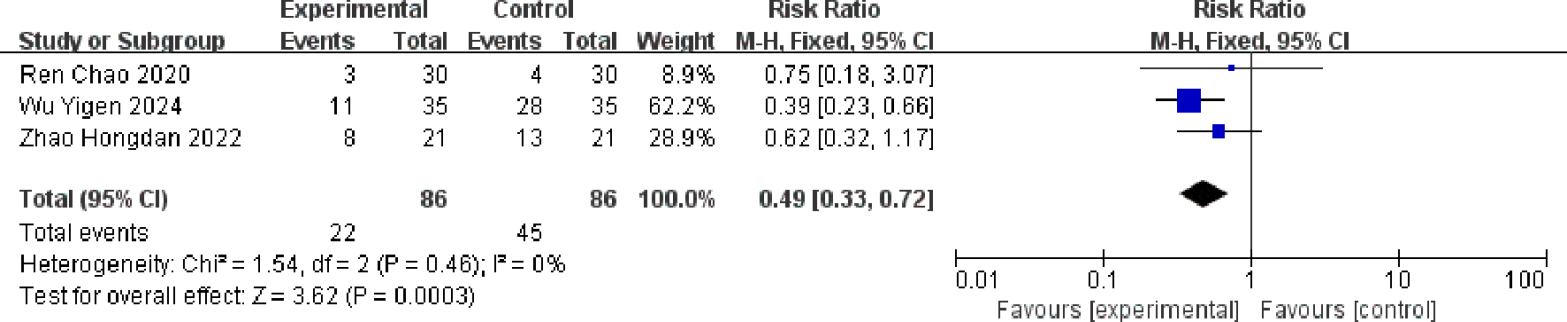
Comparison of the incidence of diarrhea between albumin-bound paclitaxel group and paclitaxel group.

Meta-analysis showed that for diarrhea, the combined effect size RR = 0.49 (0.33, 0.72), which was statistically significant (Z = 3.62, p = 0.003 < 0.05). The result of the random-effects model was RR = 0.27 (0.20, 0.35), which was statistically significant (Z = 3.66, p < 0.001). There was no statistical heterogeneity between studies (p = 0.46, I^2^ = 0%). This showed that the incidence of diarrhea in the albumin-bound paclitaxel group was 49% of that in the paclitaxel group. The forest plot for the analysis of diarrhea is shown in **Figure 10**.

**Figure 11:**
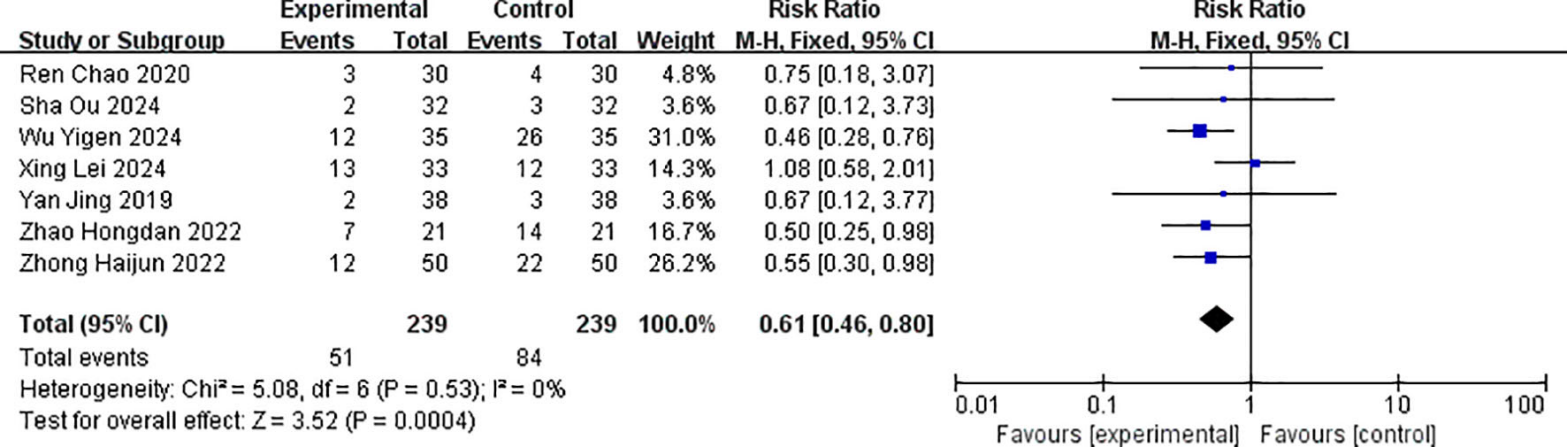
Comparison of the incidence of nausea and vomiting between albumin-bound paclitaxel group and paclitaxel group.

For nausea and vomiting, the combined effect size RR = 0.61 (0.46, 0.80), which was also statistically significant (Z = 3.52, p = 0.0004 < 0.05). The result of the random-effects model was RR = 0.60 (0.45, 0.79), which was statistically significant (Z = 3.60, p < 0.001), and there was no statistical heterogeneity between studies (p = 0.53, I^2^ = 0%). This suggested that the incidence of nausea and vomiting in the albumin-bound paclitaxel group was 61% of that in the paclitaxel group. The forest plot for the analysis of nausea and vomiting is shown in **Figure 11**.

**Figure 12:**
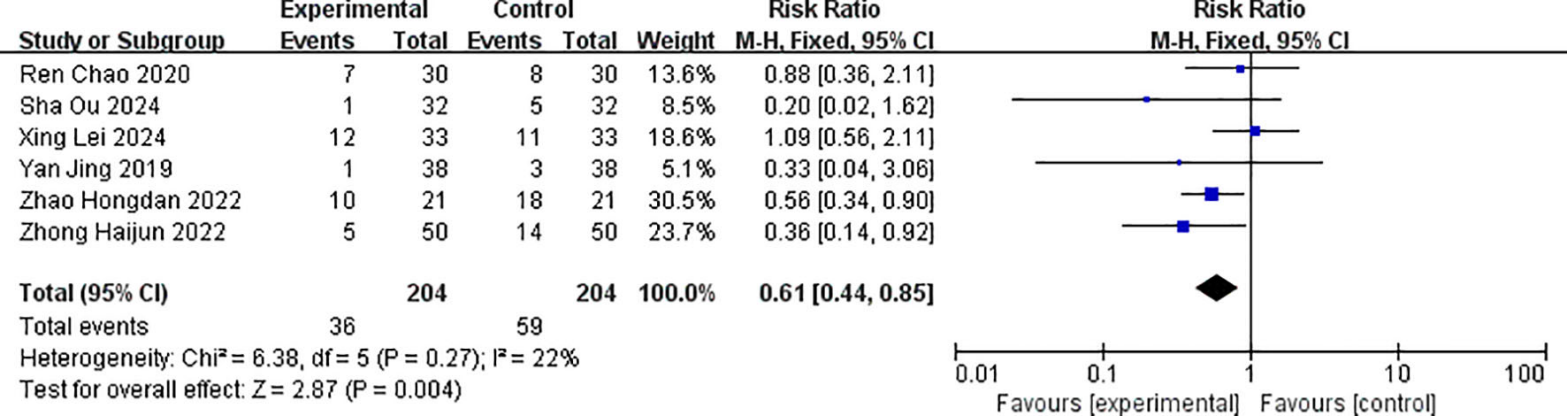
Comparison of the incidence of thrombocytopenia between albumin-bound paclitaxel group and paclitaxel group.

The combined effect size RR = 0.61 (0.44, 0.85) for thrombocytopenia, which was statistically significant (Z = 2.87, p = 0.004 < 0.05). The result of the random-effects model was RR = 0.63 (0.42, 0.94), which was statistically significant (Z = 2.24, p < 0.005), and there was no statistical heterogeneity between studies (p = 0.27, I^2^ = 22%). This showed that the incidence of thrombocytopenia in the albumin-bound paclitaxel group was 61% of that in the paclitaxel group. The forest plot for the analysis of thrombocytopenia is shown in **Figure 12**.

**Figure 13:**
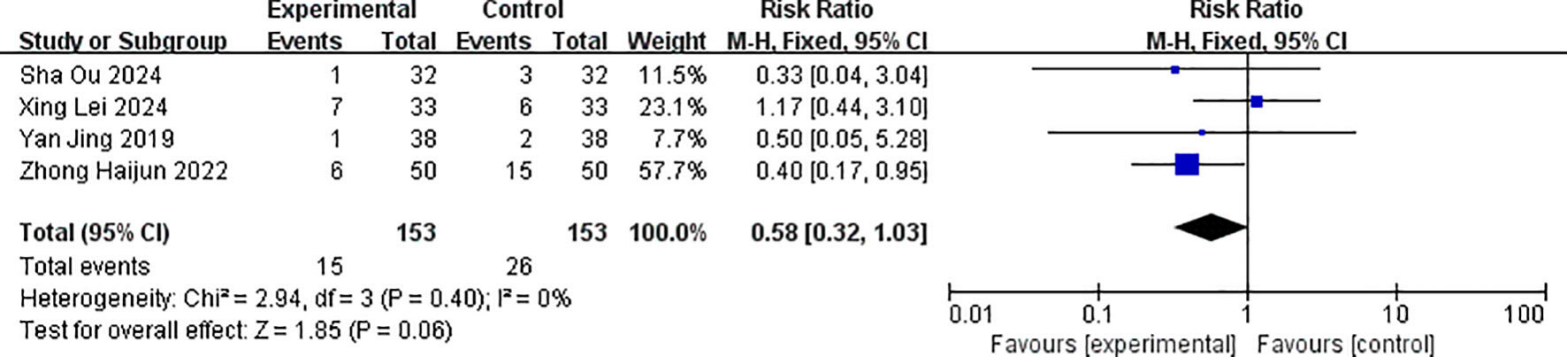
Comparison of the incidence of granulocytopenia between albumin-bound paclitaxel group and paclitaxel group.

The combined effect size RR = 0.58 (0.32, 1.03) for granulocytopenia, which was not statistically significant (Z = 1.85, p = 0.06 > 0.05). The result of the random-effects model was RR = 0.60 (0.33, 1.09), which was not statistically significant (Z = 1.67, p = 0.09 > 0.05). There was no statistical heterogeneity between studies (p = 0.4, I^2^ = 0%). This suggested that there was no significant difference in the incidence of thrombocytopenia between the albumin-bound paclitaxel group and the paclitaxel group. The forest plot for the analysis of granulocytopenia is shown in **Figure 13**.

**Figure 14:**
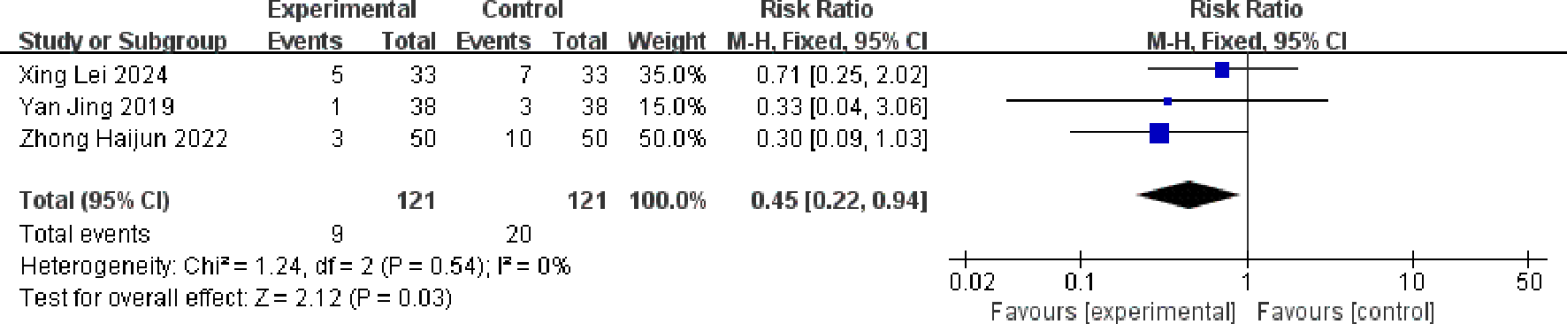
Comparison of the incidence of musculoskeletal pain between albumin-bound paclitaxel group and paclitaxel group.

For musculoskeletal pain, the combined effect size RR = 0.45 (0.22, 0.94), which was statistically significant (Z = 2.12, p = 0.03 < 0. 05). The result of the random-effects model was RR = 0.48 (0.22, 1.00), which was statistically significant (Z = 1.95, p = 0.05). There was no statistical heterogeneity between studies (p = 0.54, I^2^ = 22%), indicating that the incidence of musculoskeletal pain in the albumin-bound paclitaxel group was 45% of that in the paclitaxel group. The forest plot for the analysis of musculoskeletal pain is shown in **Figure 14**.

### 3.5 Sensitivity analysis

**Figure 15:**
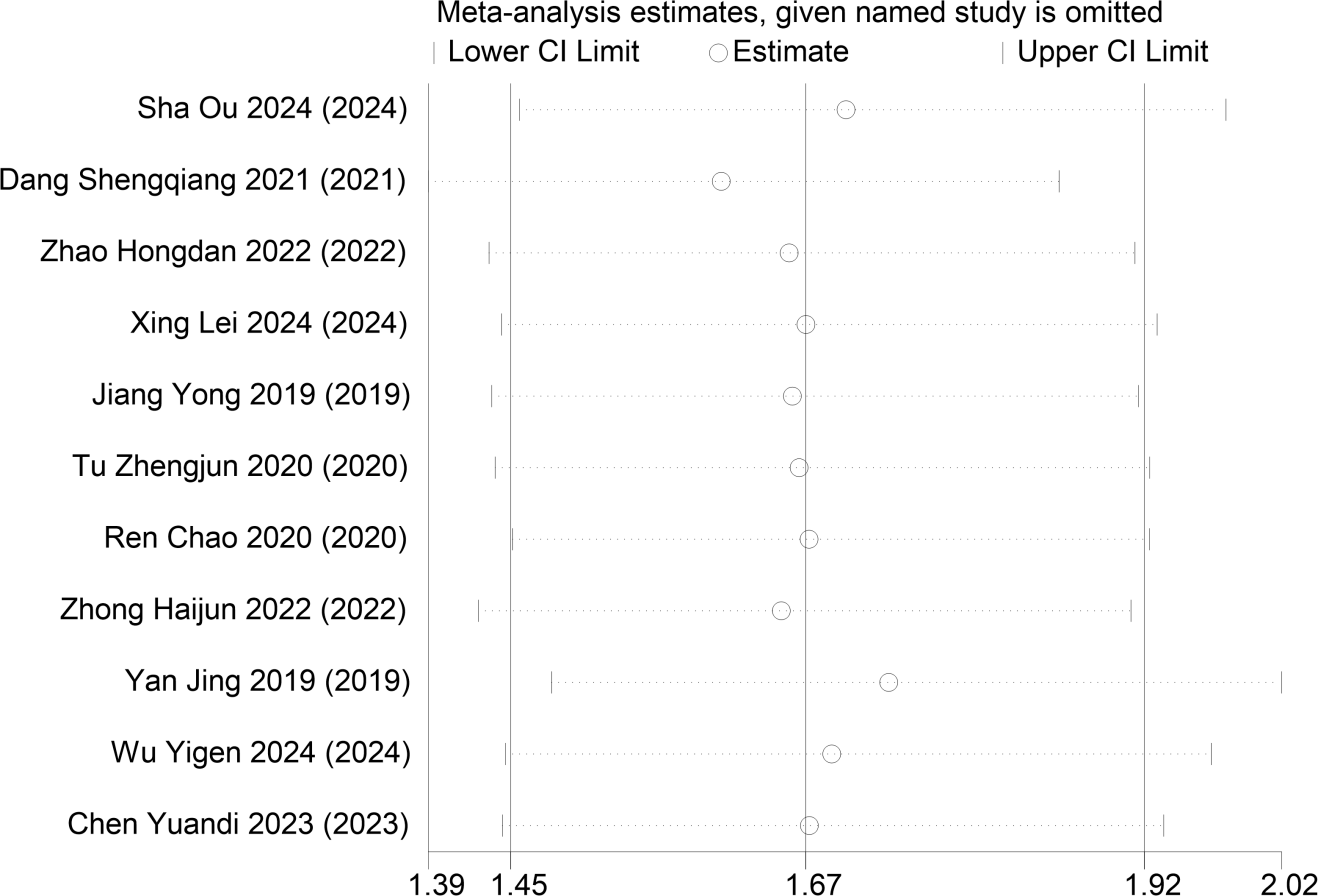
Sensitivity analysis of objective response rate.

The sensitivity analysis of objective response rate was conducted, and the results showed that the combined effect size of the remaining studies was within the 95% confidence interval, and no significant change occurred after eliminating each study. This indicated that the results of this meta-analysis were robust and reliable. The sensitivity analysis of the objective response rate is shown in **Figure 15**.

### 3.6 Bias test

**Figure 16:**
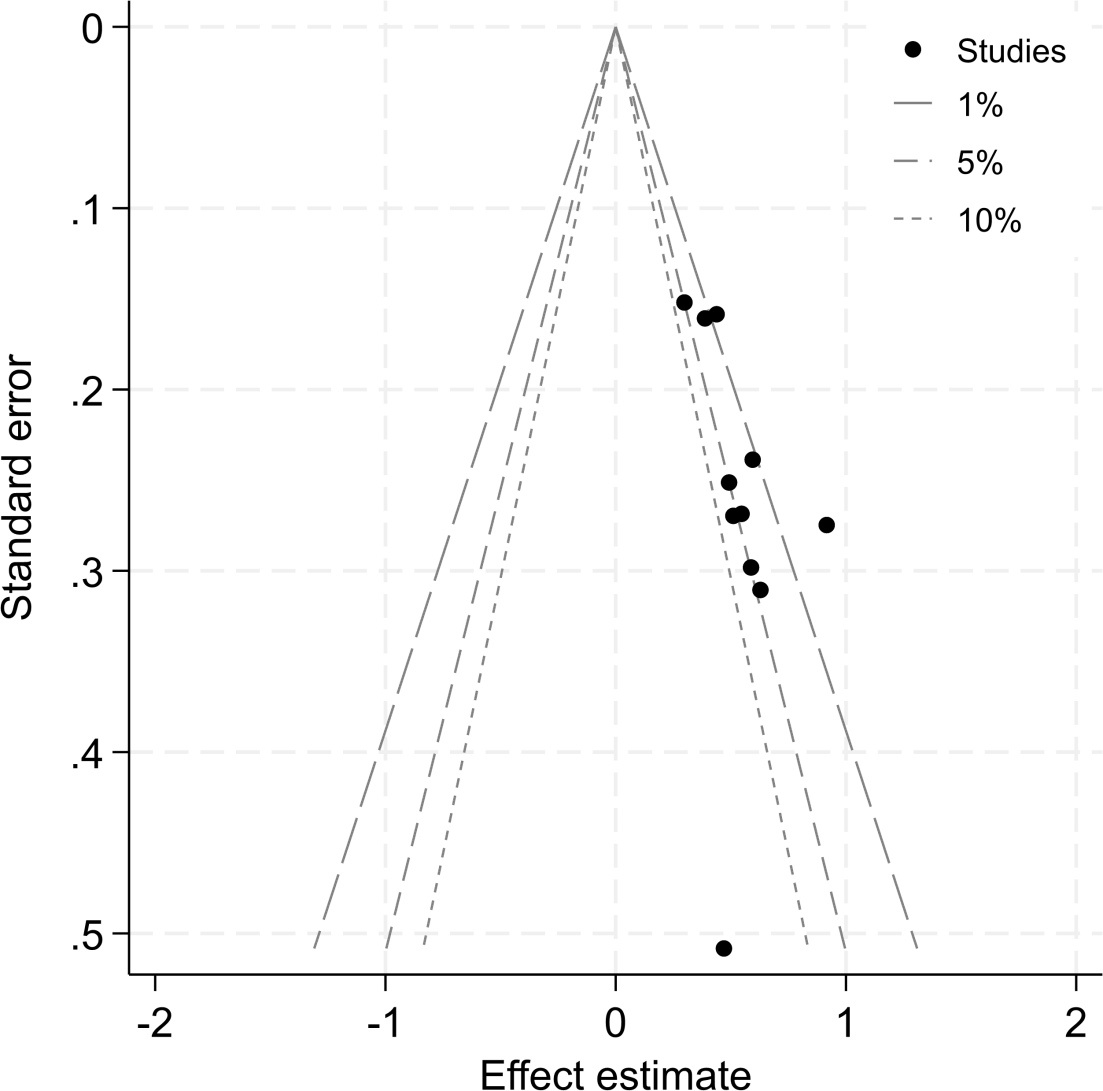
Contour-enhanced meta-analysis funnel plots of objective response rate.

Contour-enhanced meta-analysis funnel plots were used to examine whether there was publication bias in the objective response rate, one of the effective indicators obtained from the meta-analysis of 11 articles. The results showed that the contour-enhanced meta-analysis funnel plots indicated a symmetrical distribution of small-sample studies between the statistically significant zone (p < 0.05) and the non-significant zone (p > 0.05). Egger’s test result was p = 0.866 (>0.05), suggesting no significant publication bias. The contour-enhanced meta-analysis funnel plots are shown in **Figure 16**.

## 4 Conclusion and discussion

Esophageal cancer is one of the cancers with high mortality globally. In 2022, there were 224,000 new cases of esophageal cancer in China, accounting for 43.83% of global incidence (24). Paclitaxel-based drugs have emerged as common chemotherapeutic agents for esophageal cancer, with various formulations approved for market use. Due to its unique formulation process, albumin-bound paclitaxel is widely used and often replaces paclitaxel formulations in clinical practice to avoid pre-treatment. However, further data support is needed to compare the efficacy and safety of these two drugs. This study adopted a systematic review and meta-analysis approach and included 11 articles to systematically review the efficacy and safety of albumin-bound paclitaxel and paclitaxel.

The results of this study showed that albumin-bound paclitaxel had a higher objective response rate and disease control rate than paclitaxel. Serum tumor markers are continuous variables with the same outcome measures, measurement methods, and units, so MD does not need to be standardized. In terms of improvement in serum tumor markers, albumin-bound paclitaxel was more effective than paclitaxel in reducing CA125, CA199, and CEA levels, but there was no statistically significant difference in improving SCC. Regarding safety, albumin-bound paclitaxel had a lower incidence of diarrhea, nausea and vomiting, thrombocytopenia, and musculoskeletal pain compared to paclitaxel, while the incidence of neutropenia was not statistically significant. Therefore, albumin-bound paclitaxel showed better efficacy in esophageal cancer compared to paclitaxel, with fewer occurrences of diarrhea, thrombocytopenia, and musculoskeletal pain.

The systematic review and meta-analysis of more than 10 articles was tested for bias, and the contour-enhanced meta-analysis funnel plots of the objective response rate were symmetrical with no publication bias. However, it should be noted that although statistical tests did not reveal publication bias (Egger’s p = 0.866), given the limited number of studies included (n = 10), the possibility of small-sample negative results not being published cannot be completely ruled out. The sensitivity analysis of the objective response rate was conducted after eliminating each study, and the results were robust and reliable.

ORR and DCR serve as short-term efficacy endpoints; high ORR or DCR does not necessarily confer survival benefit and therefore cannot serve as surrogates for overall survival (OS) or progression-free survival (PFS). The rationale for employing ORR and DCR as primary efficacy endpoints in this study is solely attributable to the paucity of literature reporting OS/PFS as outcome measures within the included studies. Consequently, whether albumin-bound paclitaxel demonstrates a superior survival benefit over conventional paclitaxel in patients with esophageal cancer warrants further investigation.

All randomized controlled trials included in this study employed an open-label design without blinding of investigators or participants. This may introduce performance bias and exaggerate between-group differences, ultimately compromising the reliability of comparative efficacy and safety outcomes between albumin-bound paclitaxel and paclitaxel, and resulting in downgraded GRADE evidence quality.

Due to the limited number of existing literature, sensitivity analysis and bias tests were not conducted for meta-analyses with fewer than 10 articles. The robustness and publication bias of the results regarding disease control rates, serum tumor markers, and adverse reactions were not analyzed. In the subgroup analysis of serum tumor markers, the heterogeneity of literature in late-stage esophageal cancer remained high, and further exploration of the sources of heterogeneity was not possible. The heterogeneity of serum tumor markers in this study was high, which may affect the reliability of combined results. The high heterogeneity of serum tumor markers observed in this study may be associated with variations in paclitaxel dosage regimens across different studies, as well as differences in the clinical stages of enrolled patients among the included trials. Subgroup analysis showed that there was no significant heterogeneity of CA125, CA199, and CEA in the neoadjuvant treatment group, and albumin-bound paclitaxel had a better improvement effect than paclitaxel. Therefore, the interpretation of the results comparing improvements in serum tumor markers should be approached with caution and objectivity.

All patients included in this study were from China, raising concerns about potential issues related to the relative homogeneity of the patient population in terms of racial and geographic background. Consequently, the external validity of the results may be limited, particularly given potential differences in pharmacogenomics, clinical practice patterns, healthcare systems, and disease burden across international settings. Further research on a more diverse population is needed to support the broader applicability of the study results.

## Data Availability

The datasets presented in this study can be found in online repositories. The names of the repository/repositories and accession number(s) can be found in the article/**Supplementary Material**.
