# Supplementary material for: Efficacy of albumin-bound paclitaxel versus paclitaxel in esophageal cancer: a systematic review and meta-analysis
Source: Front Oncol. 2025 Aug 18;15:1612678. doi: 10.3389/fonc.2025.1612678 (PMC12399373; doi:10.3389/fonc.2025.1612678)
Supplement: Supplementary file 1 [file DataSheet1.pdf]

The English databases searched in this study included PubMed, Cochrane Library, and Embase. Since the Medline database is included in the PubMed database, it was not searched separately. The following are the search terms for the three databases:

(1)PUBMED：

**((((((((Albumin-Bound Paclitaxel[MeSH Major Topic]) OR (Protein-Bound Paclitaxel[Title/Abstract])) OR (Protein Bound Paclitaxel[Title/Abstract])) OR (ABI007[Title/Abstract])) OR (ABI-007[Title/Abstract])) OR (ABI-007[Title/Abstract])) OR (Abraxane[Title/Abstract])) AND ((((((((((Paclitaxel[MeSH Major Topic]) OR (Taxol[Title/Abstract])) OR (Bris Taxol[Title/Abstract])) OR (Taxol A[Title/Abstract])) OR (NSC?125973[Title/Abstract])) OR (Paxene[Title/Abstract])) OR (Anzatax[Title/Abstract])) OR (Onxol[Title/Abstract])) OR (7?epi?Taxol[Title/Abstract])))) AND ((((((((((Esophageal Neoplasms[MeSH Major Topic]) OR (Esophageal Neoplasm[Title/Abstract])) OR (Esophagus Neoplasm[Title/Abstract])) OR (Esophagus Neoplasms[Title/Abstract])) OR (Cancer of Esophagus[Title/Abstract])) OR (Esophageal Cancer[Title/Abstract])) OR (Esophageal Cancers[Title/Abstract])) OR (Cancer of the Esophagus[Title/Abstract])) OR (Esophagus Cancer[Title/Abstract])) OR (Esophagus Cancers[Title/Abstract]))**

(2)Cochrane Library：

| # | Searches | Results |
|---|---|---|
| 1 | MeSH descriptor: [Albumin-Bound Paclitaxel] explode all trees | 1107 |
| 2 | MeSH descriptor: [Paclitaxel] explode all trees | 5199 |
| 3 | MeSH descriptor: [Esophageal Neoplasms] explode all trees | 2715 |
| 4 | #1 AND #2 AND #3 | 57 |

（3）Embase:

| # | Searches | Results |
|---|---|---|
| 1 | 'albumin-bound paclitaxel':ti,ab,kw OR 'protein-bound paclitaxel':ti,ab,kw OR 'protein bound paclitaxel':ti,ab,kw OR abi007:ti,ab,kw OR 'abi 007':ti,ab,kw OR abraxane:ti,ab,kw | 2667 |
| 2 | 'paclitaxel'/exp | 156512 |
| 3 | paclitaxel:ti,ab,kw OR taxol:ti,ab,kw OR 'bris taxol':ti,ab,kw OR 'taxol a':ti,ab,kw OR nsc125973:ti,ab,kw OR paxene:ti,ab,kw OR anzatax:ti,ab,kw OR onxol:ti,ab,kw OR '7 epi taxol':ti,ab,kw | 79849 |
| 4 | #2 OR #3 | 163292 |
| 5 | 'esophagus tumor'/exp | 125202 |
| 6 | 'esophageal neoplasms':ti,ab,kw OR 'esophageal neoplasm':ti,ab,kw OR 'esophagus neoplasm':ti,ab,kw OR 'esophagus neoplasms':ti,ab,kw OR 'cancer of esophagus':ti,ab,kw OR 'esophageal cancer':ti,ab,kw OR 'esophageal cancers':ti,ab,kw OR 'cancer of the esophagus':ti,ab,kw OR 'esophagus cancer':ti,ab,kw OR 'esophagus cancers':ti,ab,kw | 50101 |
| 7 | #5 AND #6 | 46516 |
| 8 | #1 AND #4 AND #7 | 41 |
